# Ten Epidemiological Parameters of COVID-19: Use of Rapid Literature Review to Inform Predictive Models During the Pandemic

**DOI:** 10.3389/fpubh.2020.598547

**Published:** 2020-12-01

**Authors:** Luciana Guerra Gallo, Ana Flávia de Morais Oliveira, Amanda Amaral Abrahão, Leticia Assad Maia Sandoval, Yure Rodrigues Araújo Martins, Maria Almirón, Fabiana Sherine Ganem dos Santos, Wildo Navegantes Araújo, Maria Regina Fernandes de Oliveira, Henry Maia Peixoto

**Affiliations:** ^1^Tropical Medicine Center, University of Brasília (UnB), Brasília, Brazil; ^2^Federal Institute of Education, Science and Technology of Tocantins (Instituto Federal Do Tocantins—IFTO), Araguaína, Brazil; ^3^Pan American Health Organization (PAHO), Brasília, Brazil; ^4^Health Technology Assessment Institute (Instituto de Avaliação de Tecnologia em Saúde—IATS/Conselho Nacional de Desenvolvimento Científico e Tecnológico), Porto Alegre, Brazil

**Keywords:** coronavirus infections, review, parameters, methods, models, statistical

## Abstract

**Objective:** To describe the methods used in a rapid review of the literature and to present the main epidemiological parameters that describe the transmission of SARS-Cov-2 and the illness caused by this virus, coronavirus disease 2019 (COVID-19).

**Methods:** This is a methodological protocol that enabled a rapid review of COVID-19 epidemiological parameters.

**Findings:** The protocol consisted of the following steps: definition of scope; eligibility criteria; information sources; search strategies; selection of studies; and data extraction. Four reviewers and three supervisors conducted this review in 40 days. Of the 1,266 studies found, 65 were included, mostly observational and descriptive in content, indicating relative homogeneity as to the quality of the evidence. The variation in the basic reproduction number, between 0.48 and 14.8; and the median of the hospitalization period, between 7.5 and 20.5 days stand out as key findings.

**Conclusion:** We identified and synthesized 10 epidemiological parameters that may support predictive models and other rapid reviews to inform modeling of this and other future public health emergencies.

## Introduction

Public Health is confronted with the challenge of protecting poulations from emerging and reemerging diseases. Among the viruses capable of causing pandemics, special prominence is given to the family Coronaviridae (1–3). These viruses are responsible for three recent major epidemics: in 2009, the Severe Acute Respiratory Syndrome (SARS), caused by the Severe Acute Respiratory Syndrome Coronavirus (SARS-CoV); in 2012, the Middle East Respiratory Syndrome, caused by the Middle East Respiratory Syndrome Coronavirus (MERS-CoV) (4); and, in 2019, the Corona Virus Disease−19 (COVID-19), caused by the Severe Acute Respiratory Syndrome Coronavirus 2 (SARS-CoV-2) (5). However, SARS-CoV-2 has peculiar clinical and epidemiological characteristics when compared with SARS-CoV, MERS-CoV, or others of the same family. These characteristics are reflected in the exponentially increasing numbers of COVID-19-related deaths (6).

The current epidemic goes back to December 31, 2019, when a pneumonia outbreak was reported in Wuhan, China, with 27 cases that were later identified as COVID-19 cases (7). In the following months, the epidemic evolved from a local problem to a pandemic with catastrophic consequences. As of August 13, ~20,5 million cases and 744,500 deaths had been reported to the World Health Organization (WHO), in all age ranges and nearly all continents—except Antarctica. The Americas are currently regarded as the epicenter of the pandemic, where 53.6% of the total recorded cases have been reported−54.7% of the cases recorded within the last 24 h in the world. The United States and Brazil are particularly affected. These countries have 5,094,500 cases (163,340 deaths) and 3,109,630 cases (103,026 deaths), respectively (8).

Understanding the parameters that influence the course of an epidemic is key for health-related decision-making and allows for planning of strategies to mitigate and control diseases, as well as provision of care to those infected and sick. The high transmissibility and virulence of SARS-CoV-2, lead to a significant rate of severe and critical cases requiring specialized care and intensive care beds, creates the need for predictive models capable of estimating health care demands and support decision-making (9–11).

Mathematical models are simplifications of complex processes involved in disease dynamics, which can lead to different results based on the method, assumptions and parameters adopted (12). To minimize uncertainties, parameters feeding the model must be valid, accurate, generalizable, and reliable, as well as adaptable in population-based terms. In an emergent situation these models may contain a series of uncertainties, due to the incipient availability of epidemiological characteristics (10, 11). This requires constant review of parameters as new information arises, as well as an ongoing literature review.

The COVID-19 emergency has prompted researchers to work toward describing different aspects of disease transmission and evolution. As a result, a significant number of scientific publications are being released daily, and the MEDLINE database alone already had 16,000 publications (keyword “COVID-19”) as of May 26, 2020—when this study was performed. Information from these publications can help decision-makers develop policies throughout the course of the emergency. However, due to the large number of studies available, identifying the relevant evidence in due time presents a great challenge and requires that the methods used in traditional literature reviews be adapted.

To support evidence-based decision-making using predictive models for the COVID-19 public health emergency, while the epidemic was establishing in Brazil, a rapid literature review method was proposed with the view to identify and describe clinical and epidemiological parameters relative to infection by SARS-CoV-2 and the illness caused by this virus, coronavirus disease 2019 (COVID-19). This article, therefore, aims to describe the methods employed in this rapid literature review and present the main epidemiological parameters describing SARS-CoV-2 transmission and the COVID-19 disease.

## Materials and Methods

A methodological proposal for rapid review of epidemiological parameters and their application in the context of the current SARS-CoV-2 pandemic emergency.

### Proposed Methodology

A rapid literature review, with the aim to identify clinical and epidemiological parameters to support mathematical models of COVID-19 transmission and disease. The proposed rapid review method developed by the authors includes the following steps: research scope definition; eligibility criteria; information sources; database search strategies; study selection; and data extraction. For method construction, we met with the group of modelers to identify the required parameters. The parameters defined for the search and their descriptions are provided in Table 1.

**Table 1:** Description of identified epidemiological parameters of COVID-19.

**Parameters**	**Description**
Basic reproduction number (R0)	The mean number of new infections arising from one infected person in a totally susceptible population (13).
Serial interval	Time between onset of symptoms in a primary case (infector) and onset of symptoms in a secondary case (infectee) (14).
Incubation period	Time between infection and onset of disease (15).
Transmissibility period	Time during which a person infected with SARS-*CoV*-*2* transmits the virus to other people.
Proportion of detected cases	Proportion of cases identified as infected with SARS-*CoV*-*2* among all cases tested.
Proportion of critical cases among hospitalized patients	Proportion of critical cases of COVID-19 among all hospitalized patients.
Proportion of deaths among critical cases	Proportion of deaths from COVID-19 among all critical cases of the disease.
Mean or median length of hospital stay	Time in days (mean or median) of hospital stay among COVID-19 cases.
Mean or median time between admission to hospital and onset of ARDS^a^	Time in days (mean or median) of hospital stay among COVID-19 cases before onset of ARDS^a^.
Length of hospital stay in wards before admission at ICU^b^	Time in days (mean or median) of hospital stay in wards among COVID-19 cases who required ICU^b^.

a *Acute respiratory distress syndrome*.

b *Intensive care unit*.

## Results

### Methodological Protocol

During the preparation stage, the group developed a methodological protocol to guide construction of the methods employed in the rapid literature review. The protocol was composed of six stages, the respective descriptions of which are provided in Table 2.

**Table 2:** Methodological proposal for quick literature review: identification of epidemiological parameters.

**Steps**	**Description**
Search scope	It should be structured as follows: definition of the population to be studied; choice of epidemiological parameters; organizing groups of parameters according to similarity (e.g., types of studies that generate them).
Eligibility criteria	For a quick and reliable selection, only include studies published as from the date of the first outbreak of the disease in the world; presenting at least one of the parameters assessed in the abstract; original investigations, literature reviews; published in English or in other languages of the group domain; including studies published in other languages, but with the abstract in the languages of the domain that allow clear identification of any parameters of interest. It is suggested for reviewers to exclude: studies from preprint databases that analyzed primary data and have not been submitted to ethical evaluation; opinion articles; epidemiological bulletins with overlapping data of the same place, and studies that do not allow a reliable translation.
Sources of information	Literature search should be divided into two phases: the first should search at least two international databases, and the second should track the lists of references of studies identified in the first stage.
Search in the databases	The search syntax must represent the problem to be investigated, its primary endpoints and the date that best represents the beginning of the first outbreak in the world. For example: (name of the disease OR name of virus) AND (endpoint 1 OR endpoint 2) AND (start date AND final date).
Study selection	Study selection should comprise the following stages: selection of studies for complete assessing, from evaluation of titles and abstracts as per eligibility criteria; reading of full texts and new evaluation considering eligibility. Non-matching stages, but with the support of a more experienced researcher to clarify doubts and organize the process.
Data extraction	Data extraction should be guided by means of a structured tool, which allows the objective identification of parameters and a quick assessment of the quality of studies, in terms of validation and accuracy of data. Non-matching stage but overseen by a researcher with a trained in epidemiology.

### Operationalizing the Rapid Literature Review

The population of interest was composed of people living in high-risk areas of SARS-CoV-2 infection. The epidemiological parameters were divided into two groups, for better organization of the syntax and database search. The first group, referred to as Group 1, included the following parameters: basic reproduction number (R0); serial interval; incubation period; transmissibility period. The second, referred to as Group 2, included the following parameters: rate of detected cases; rate of critical cases among all hospitalized patients; rate of deaths among critical cases; mean or median length of hospital stay; mean or median time between hospital admission and ARDS (Acute Respiratory Distress Syndrome) onset; or mean or median length of hospital stay before ICU (Intensive Care Unit) admission.

To identify Group 1 and Group 2 parameters, we selected studies indexed in databases: Medical Literature Analysis and Retrieval System Online *(*MEDLINE) and Excerpta Medica dataBASE (EMBASE). For each group of parameters, we organized search syntaxes on MEDLINE, via PubMed and on EMBASE, based, respectively, on MeSH (Medical Subject Headings) and Emtree (Embase Subject Headings) terms. Searches were performed in two stages, one on March 27, 2020 and the second on April 13, 2020. Additional studies were obtained from mannualy searches in the references of the selected articles and reviews.

We organized four search syntaxes based on the group of parameters and the database. Table 3 shows the search syntaxes used to identify studies on MEDLINE via PubMED, which were adapted for EMBASE. Duplicates were removed with the help of reference management software programs Mendeley Desktop version 1.19.4 and Covidence.

**Table 3:** Search syntax: MEDLINE.

**MEDLINE/GROUP 1:** ((“coronavirus”[MeSH Terms] OR “severe acute respiratory syndrome coronavirus 2”[Supplementary Concept] OR “severe acute respiratory syndrome coronavirus 2”[All Fields]) OR “COVID-19”[All Fields] OR “COVID-19”[Supplementary Concept] OR “Novel Coronavirus” [All Fields]) AND ((((“basic reproduction number” [MeSH Terms] OR (“basic” [All Fields] AND “reproduction”[All Fields] AND “number”[All Fields]) OR “basic reproduction number”[All Fields]) OR R0[All Fields] OR “basic reproductive number”[All Fields] OR (basic[All Fields] AND (“reproduction”[MeSH Terms] OR “reproduction”[All Fields] OR “reproductive”[All Fields]) AND number[All Fields])) OR (“infectious disease incubation period”[MeSH Terms] OR (“infectious”[All Fields] AND “disease”[All Fields] AND “incubation”[All Fields] AND “period”[All Fields]) OR “infectious disease incubation period”[All Fields])) OR ((“disease transmission, infectious”[MeSH Terms] OR (“disease”[All Fields] AND “transmission”[All Fields] AND “infectious”[All Fields]) OR “infectious disease transmission”[All Fields] OR (“disease”[All Fields] AND “transmission”[All Fields] AND “infectious”[All Fields]) OR “disease transmission, infectious”[All Fields]) OR (communicable[All Fields] AND period[All Fields]))) AND (“2020/01/01”[PDAT] : “2020/04/13”[PDAT])
**MEDLINE/GROUP 2:** ((“coronavirus”[MeSH Terms] OR “severe acute respiratory syndrome coronavirus 2”[Supplementary Concept] OR “severe acute respiratory syndrome coronavirus 2”[All Fields]) OR “COVID-19”[All Fields] OR “COVID-19”[Supplementary Concept] OR “Novel Coronavirus”[All Fields]) AND ((((((((“Hospitalization”[Mesh] OR “Critical Care”[Mesh]) OR “Intensive Care Units”[Mesh]) OR “Critical Illness”[Mesh]) OR “Mortality”[Mesh]) OR “Hospital Mortality”[Mesh]) OR “Pneumonia, Ventilator-Associated”[Mesh]) OR “Disease Attributes”[Mesh] OR “hospitalized patient*”[All Fields] OR (epidemiological characteristic[All Fields] OR epidemiological characteristics[All Fields]) OR (clinical characteristic[All Fields] OR clinical characteristics[All Fields]) OR (clinical outcome[All Fields] OR clinical outcomes[All Fields] OR clinical outcomes,[All Fields]) OR “hospitalization rate”[All Fields] OR (undocumented[All Fields] AND (“infections”[MeSH Terms] OR “infections”[All Fields] OR “infection”[All Fields])) OR “documented infection”[All Fields]) OR (((((((“Epidemiologic Studies”[Mesh] OR “Clinical Studies as Topic”[Mesh]) OR “Epidemiologic Study Characteristics”[Mesh]) OR “Decision Support Techniques”[Mesh]) OR “Case Reports”[Publication Type]) OR “Observational Study”[Publication Type]) OR “Cohort Studies”[Mesh]) OR ((“IEEE Int Conf Automation Sci Eng (CASE)”[Journal] OR “CASE (Phila)”[Journal] OR “case”[All Fields]) AND (“SERIEs (Berl)”[Journal] OR “series”[All Fields])))) AND (“2020/01/01”[PDAT] : “2020/12/31”[PDAT]) AND (“2020/01/01”[PDAT] : “2020/04/13”[PDAT])

The eligibility criteria included studies published as of January 1, 2020. We included original research studies, epidemiological bulletins and literature reviews addressing any of the parameters of interest, published in English, Spanish or Portuguese. Studies in other languages were included only when any of the parameters of interest could be identified in the Abstract published in English, Spanish or Portuguese. The list of elegibility criteria is presented in Table 2.

For study selection, the titles and abstracts identified were classified as per the inclusion and exclusion criteria. Studies that met the inclusion criteria and none of the exclusion criteria were selected for full reading and reassessed for eligibility. Data were extracted based on three spreadsheets specifically developed for the parameters addressed in the review.

Data search, inclusion, reading, and extraction were not conducted in a paired fashion, and each study was reviewed by an investigator under supervision by a second, more experienced investigator with an epidemiology background. The supervisor supported every stage of the review, providing guidance and answering questions, and that data extraction was entirely verified by two supervisors.

### Epidemiological Parameters

**Figure 1:**
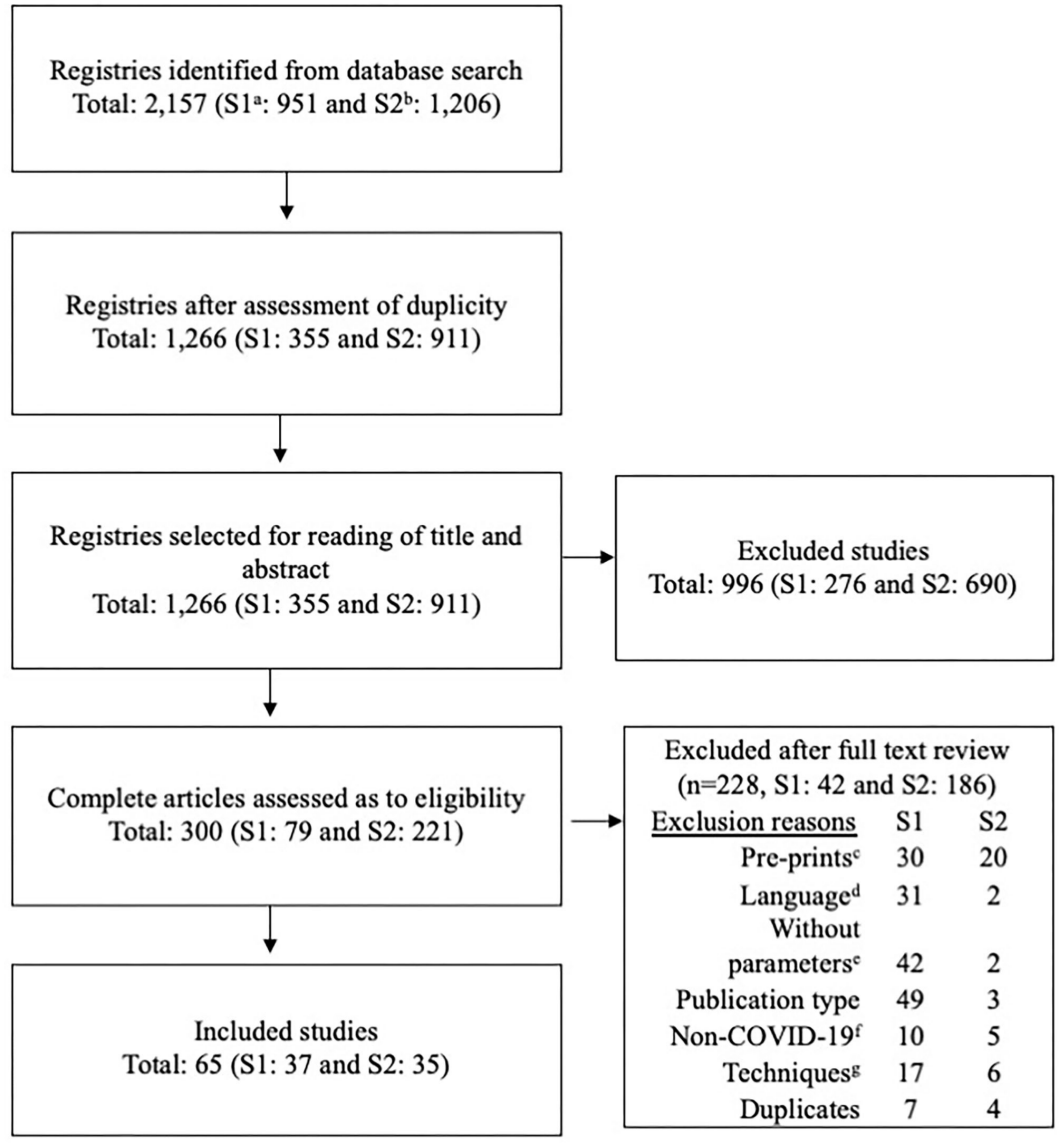
Flowchart of the selection process of evidence of clinical and epidemiological parameters of COVID-19. a, First group of parameters (syntax group 1); b, Second group of parameters (syntax group 2); c, articles published as pre-prints; d, article in non-English, Spanish, or Portuguese and the parameter data was not included in the abstract; e, it was not possible to extract the parameters of interest; f, did not provide data on COVID-19; g, Laboratory studies or other techniques.

Figure 1 describes the flow of information at different stages of the review. At first, we found 951 studies using the strategies set up to identify parameters in Group 1 and 1,206 studies using the strategies to retrieve parameters in Group 2. After assessing for duplicates, we were left with 1,266 studies (Group 1: 355 and Group 2: 911), of which 65 were included (Group 1: 37 and Group 2: 35).

Epidemiological parameters were divided into 3 datasets according to the groups searched.

Table 4 shows search results by basic reproduction number (R0) and time-varying reproduction number (Rt)—when present—in the 19 studies identified (16–18, 20–35). Analyses relied mostly on data from China, followed by Japan and South Korea, and were performed between December 2019 and March 2020. The highest R0 identified was 14.8, estimated for the Diamond Princess cruise-ship during its quarantine in Japan (22), and the lowest was 0.48 - in South Korea (30). Decreases in SARS-CoV-2 reproduction numbers have been seen after restrictive measures were implemented.

**Table 4:** Presentation of parameters: basic reproduction number (R0).

**Author, year [references]**	**Country**	**Date of study**	**Method:**	**R0^a^**	**Others**
Chen et al., 2020 (16)	China	Between December 2019 and January 2020	Reservoir-people transmission network model^c^	3,58.	–
Chen et al., 2020 (16)	Japan (Diamond Princess cruise ship)	February 2020	Package “earlyR”	2.28 (95%CI: 2.06–2.52)	–
Kuniya, 2020 (17)	Japan	Between January and February 2020	SEIR Model^d^	2.6 (95%CI: 2.4–2.8)	–
Fang et al., 2020 (18)	China	Between January and February 2020	SEIR Model^d^	–	R according to date: Day 0: 2.4; day 10: 3.2; day 20: 2.98, etc.
Zhao et al., 2020 (19)	China	January 2020	Statistical exponential growth model^e^	2.24 (95%CI: 1.96—2.55)	–
Kucharski et al., 2020 (20)	China	Between January and February 2020	Stochastic transmission model	–	Rt^b^(before and after restriction measures): 2.35 (95%CI: 1.15–4.77) to 1.05 (95%CI: 0.41–2.39)
Wang et al., 2020 (21)	China	Between December 2019 and February 2020	SEIR Model^d^	3.1	Rt^b^ measured in distinct phases after restriction measures: 2.6; 1.9 and 0.5
Rocklöv et al., 2020 (22)	Japan (Diamond Princess cruise ship)	January 2020	SEIR Model^d^	14.8	Rt^b^ after restriction measures: 1.74
Mizumoto and Chowell, 2020 (23)	Japan (Diamond Princess cruise ship)	Between January and February 2020	Next generation matrix (NGM)^f^ in a totally susceptible population		Maximum Rt^b^: 11.2 (95%CI: 7.5—16.2) and median Rt: 5.8 (95%CI: 0.6–11.0)
Tang et al., 2020 (24)	China	January 2020	SEIR Model^d^	6.47 (95%CI: 5.71—7.23)	–
Wu et al., 2020 (25)	China	Between December 2019 and January 2020	SEIR Model^d^	2.68 (95%CI: 2.47–2.86)	–
Li et al., 2020 (26)	China	January 2020	Statistical exponential growth model^e^	2.2 (95%CI: 1.4–3.9).	–
Zhao et al., 2020 (27)	China	January 2020	Exponential growth model by Poisson in a completely susceptible population	2.56 (95%CI: 2.49–2.63)	–
Wang et al., 2020 (28)	China	Between January and February 2020	Statistical exponential growth model^e^	3.49 (95%CI: 3.42–3.58)	After containment measures Rt^b^: 2.95 (95%CI: 2.86–3.03)
Zhou et al., 2020 (29)	China	January 2020	SEIR Model^d^	2.8–3.3	–
Ki and Task Force for 2019-nCoV, 2020 (30)	South Korea	Between January and February 2020	Not informed	0.48 (95%CI: 0.25–0.84)	–
Du et al., 2020 (31)	China	Between January and February 2020	Exponential growth model based on public data from Wuhan	1.32 (95%CI: 1.16–1.48)	–
Anastassopoulou et al., 2020 (32)	China	Between January and February 2020	SIDR model^g^	2–2.6	Other R0^a^ estimates based on linear regression varied between 3.2 (95%CI: 2.4–4) and 5.14 (95%CI: 4.25–6.03).
Song et al., 2020 (33)^*^	China	January 2020	Weibull distribution methods, Gamma Lognormal,	Weibull distribution methods: 3.74 (95%CI: 3.63–3.87); Gamma: 3.16 (95%CI: 2.90–3.43); Lognormal: 3.91 (95%CI: 3.71–4.11)	–
Pan et al., 2020 (34)	China	Between December 2019 and March 2020	Used a method proposed by Cori et al. (2013)^h^ in R version 3.6.2.	–	Maximum Rt^b^ — 03/01/2020: 3.94 (95%CI: 3.32—-4.63); Minimum Rt^b^: 08/03/2020: 0.10 (95%CI: 0.08—-0.13)

a *R0, basic reproduction number*.

b *Rt, effective basic reproduction number (variable in time)*.

c *Reservoir–People transmission network*.

d *SEIR: Susceptible – Exposed – Infected – Recovered*.

e *Statistical exponential growth model*.

f *NGM: next–generation matrix*.

g *SIDR: Susceptible – Infected – Recovered – Death*.

h *Cori A et al. (2013). doi: 10.1093/aje/kwt133*.

* *Article in Chinese, only the abstract in English was assessed*.

Data extracted from studies on incubation and transmissibility periods and serial interval can be seen in Table 5. We identified 22 studies (14, 15, 26, 30, 31, 33, 36–51) of which 19 (14, 15, 26, 30, 33, 36–41, 43, 44, 46–51) addressed the incubation period, with means ranging from 3.6 days (48) to 6.7 days (39); one addressed the transmissibility period [Median: 9.5 days, interquartile range (IQR) 3.5–13.0] (42); and five (26, 30, 31, 36, 45) addressed the serial interval, with means ranging from 3.96 (31) to 7.5 days (26). The parameters presented in the chart were predominantly estimated based on descriptive observational studies from China, South Korea, and Singapore.

**Table 5:** Presentation of serial interval parameters, incubation period, and transmissibility period.

**Author, year [references]**	**Country**	**Study date**	**Characteristics of the sample (sample, age, and sex)**	**Incubation period**	**Transmissibility period**	**Serial interval**
Linton et al., 2020 (14)	China	January 2020	*N* = 158 cases; predominance of age group 30–39 years; Female sex: 42%	Mean: 5.6. (95%CI: 5.0–6.3 days).	–	–
Song et al., 2020 (33)^*^	China	January 2020	No information	Mean: 5.01. (95%CI: 4.31–5.69 days).	–	–
Pung et al., 2020 (36)	Singapore	February 2020	*N* = 36 cases; 17 positive cases; Age [median (IQR)]: 40 (36–51) years; Female sex: 59%	Median: 4 days (IQR: 3–6 days).	–	Range: 3–8 days
Chan et al., 2020 (37)	China	January 2020	*N* = 6 cases; 5 adults and one child, Age (mean): 45 years; Female sex: 50%	Range: 3–6 days	–	–
Lauer et al., 2020 (38)	50 places in and outside China	January and February 2020	*N* = 181 cases; Age [median (IQR)]: 44.5 (34–55,5) years; Female sex: 38%	Median: 5.1 days (95%CI: 4.5–5.8 days).	–	–
Fan et al., 2020 (39)	China	January and February 2020	*N* = 54 cases; Age [mean (lower and upper limits)]: 38 (1, 67–94) years; Female sex: 33.3%; 19 cases subsidized the estimated incubation period	Mean: 6.7 days	–	–
Yang et al., 2020 (40)^*^	China	January and February 2020	*N* = 325 cases	Median: 7 days; Mode: 4 days Range: 1–20 days	–	–
Backer et al., 2020 (41)	China	January 2020	*N* = 88 cases; Age (lower and upper limits): 2–72 years; Female sex: 35.2%	Mean: 6.4 days (95%CI: 5.6–7.7 days).	–	–
Hu et al., 2020 (42)	China	January and February 2020	*N* = 24 cases; Age [median (IQR)]: 32.5 (19.0–57.0) years; Female sex: 77.7%	–	Median: 9.5 days (IQR 3.5–13.0 days)	
Li et al., 2020 (26)	China	January 2020	*N* = 425 cases; Age [median (lower and upper limits)]: 59 (15–89) years; Female sex: 49%; 10 cases subsidized the estimated incubation period, and 6 pairs subsidized the serial interval calculation	Mean: 5.2 days (95%CI: 4.1–7.0 days)	–	Mean (*SD*): 7.5(±3.4) days (95%CI: 5.3–19 days)
Wu et al., 2020 (43)^*^	China	January 2020	*N* = 40 cases; 35% of the aged over 60 years	Median: 6 days	–	–
Zhang et al., 2020 (44)^*^	China	January 2020	*N* = 17 cases; Age [median (lower and upper limits)]: 55 (19–79) years; Female sex: 23.5%	Median: 4 days Range: 0–12 days	–	–
Ki and Task Force for 2019-nCoV, 2020 (30)	South Korea	January and February 2020	*N* = 28 cases; Age [median (lower and upper limits)]: 42 (21–73) years; Female sex: 46.4%	Mean: 3.9 days Median: 3 days	–	Mean: 6.6 days; Median: 4 days
Nishiura et al., 2020 (45)	China	February 2020	Data from articles and reports: 28 pairs.	–	–	Median: 4.6 days (95%CI: 3.5–5.9 days)
Lian et al., 2020 (46)	China	Between January 17 and February 12, 2020	*N* = 778 cases, divided into elderly group, with 136 cases and mean age of 68.28 years; and non-elderly adult group with 652 cases and mean age of 41.15 years.	Median: 5 days (IQR: 2–9 days)	–	–
Sun et al., 2020 (15)	China	January 2020	*N* = 33 cases	Median: 4.5 days (IQR: 0–5.5 days) Maximum: 9.5 days	–	–
Jia et al., 2020 (47)	China	Between January and February 2020	*N* = 44 cases; Age [median (lower and upper limits)]: 46 (1 and 90 years); Female sex: 65.9%	Mean: 6.28 days Range: 1–14 days	–	–
Du et al., 2020 (31)	China	Between January and February 2020	*N* = 468 cases	–	–	Mean: 3.96 (95%CI: 3.53–4.39 days) *SD* ± 4.75 days (95%CI: 4.46–5.07 days)
Guan et al., 2020 (48)	China	Between December 2019 and January 2020	*N* = 1,590 hospitalized patients; Mean age: 48.9 (*SD* ± 16.3) years; Female sex: 42.7%	Mean: 3.6 days (*SD* ± 4.2 days)	–	–
Jin et al., 2020 (49)	China	Between January and February 2020	*N* = 651, divided into group with gastrointestinal symptoms (GI) (*N* = 21), Age [mean (*SD*)]: 46.14 ± 14.19 years; Female sex: 50%; and asymptomatic group GI (*N* = 577), Age [mean (*SD*)]: 45.09 ± 14.45 years; Female sex: 49.05%	With GI symptoms: median: 4 days (IQR: 3–7 days). With no GI symptoms: median: 5 days (IQR: 3–8 days)	–	–
Sun et al., 2020 (50)	China	Between January and February 2020	*N* = 8 children; Age (lower and upper limits): 2 months to 15 years; Female sex: 25%	Range: 5–10 days	–	–
Guan et al., 2020 (51)	China	Up to January 2020	*N* = 1,099; Age (median): 47 (IQR 35–58) years; Female sex: 41.9%	Median: 4 days (IQR: 2–7 days)	–	–

*N: total number of participants in the study*.

*GI: gastrointestinal symptoms*.

*95%CI: 95% confidence interval*.

*IQR: Interquartile range*.

*SD: Standard deviation*.

* *Article in Chinese, only the abstract in English was assessed*.

Table 6 shows results for Group 2 and the rate of detected cases; rate of critical cases among all hospitalized patients; rate of deaths among critical cases; mean or median length of hospital stay; mean or median time between hospital admission and ARDS onset; or mean or median length of hospital stay before ICU admission. These parameters were mostly extracted from studies in China, predominantly with adult and elderly subjects aged between 41 and 68 years, and males. Of the 35 studies reviewed (34, 46, 49–79), only three (52, 53, 79) showed the proportion of cases identified as infected with SARS-CoV-2 among all cases tested (detected cases), and numbers ranged between 4.45% (79) and 61.8% (53). In the studies, there was variability in the criteria used to define cases as critical, and data showed that the proportion of critical COVID-19 cases among all hospitalized patients ranged between 0.06% (51) and 86.9% (56). Not all studies reported the number of deaths among critical cases, and when they did (49, 51, 56, 57, 60–62, 65–67, 70, 73, 75, 77, 78), the rate identified was 1.35% (49) to 78% (60). The median length of total stay among COVID-19 cases ranged from a minimum of 7.5 days (cases with a death outcome) (60) and a maximum of 20.5 days (77). The median length of outpatient stays prior to ARDS onset ranged between two (66) and 14 (62) days. The median lenght of outpatients stays prior to ICU admission was reported in one study as one day (57) and the mean as 8 days (64).

**Table 6:** Presentation of parameters: detected cases, critical cases among hospitalized patients, deaths among the critical cases, hospitalization period, and hospitalization period before ARDS or ICU.

**Author, year [references]**	**Country**	**Study date**	**Characteristics of the sample (sample, age, and sex)**	**Cases detected N/D (%)**	**Critical cases among hospitalized patients N/D (%)**	**Deaths among critical cases**	**Total length of stay (*N*)—mean/median**	**Period between hospitalization before ARDS OR ICU (*N*)—mean/median**
R. Li et al., 2020 (52)	China	March 2020	No information	?/? (14%; 95%CI: 10–18%)^a^	–	-	–	–
Novel Coronavirus Pneumonia Emergency Response Epidemiology Team, 2020 (53)^*^	China	February 2020	*N* = 672 cases; 86.6% aged between 30 and 79 years	44,672 /72,314 (61.8%)	–	-	–	-
Zheng et al., 2020 (54)	China	February 2020	*N* = 25 cases; Age [median (IQR; range)]: 3 years (2–9 years; 3 months−14 years); Female sex: 44%	–	2^b^/25 (8%)	–	–	–
Peng et al., 2020 (55)^*^	China	Between January and February 2020	*N* = 112 cases; adults with cardiovascular disease, age (mean): 62 years; Female sex: 52.67%	–	16^c^/ 112 (14.28%)	–	–	–
Liu et al., 2020 (56)	China	Between December 2019 and January 2020	*N* = 137 hospitalized patients; Age [median (lower and upper limits)]: 57 (20–83) years: Female sex: 55.47%	–	119^b^/137 (86.9%)	16/137 (11.7%)	–	(137)—Median of 7 days, ranging from 1 to 20 days – ARDS^*g*^
Wang et al., 2020 (57)	China	Between January and February 2020	*N* = 138 hospitalized patients; Age [median (IQR; lower and upper limits)]: 56 (42–68; 22–92) years: Female sex: 45, 7%	–	36^c^/138 (26.1%)	6/36 (16.66%)	(47)—Median (among those who were discharged): 10 days (IQR 7–14 days)	(138)—Median: 5 days (IQR 1-10 days)^g^ median of 1 day (IQR 0 – 3 days)^c^
Cheng et al., 2020 (58)^*^	China	Up to February 2020	*N* = 1,079 cases; Age [mean (±*SD*)]: 46 (24) years: Female sex: 46, 8%	–	72^d^/ 1265 (5.7%)	–	–	–
Guan et al., 2020 (51)	China	Up to January 2020.	*N* = 1,099 cases; Age (median): 47 (IQR 35–58) years; Female sex: 41.9%	–	67^c^/1,099 (0.06%)	15/173 (8.67%)	Median of 13 (IQR 11.5–17.0 days)	Median of 5 days (IQR 2–7 days) between onset of symptoms and onset of pneumonia
Mo et al., 2020 (59)	China	Between January and February 2020	*N* = 155 hospitalized patients; Age [median (IQR)]: 54 (42–66) years; Female sex: 44.50%	–	37^e^/ 155 (23.9%)	–	(22)—Median of fatal cases: 10.5 (IQR: 8–16 days); 133-−10 recovered cases (IQR 7–15 days)	-
Zhou et al., 2020 (60)	China	Up to January 2020	*N* = 191 hospitalized patients; Age [median (IQR)]: 56 (46–67) years; Female sex: 38%	–	53^e^/191 (28%)	42/53 (78%)	(191)—median: 11 days (IQR: 7–14 days)^k^; (54)—Fatal cases: 7.5 (IQR: 5–11 days); (137)—Survivors: 12 (IQR: 9–15 days)	(191)—median: 12 days (IQR: 8-−15 days)^g^
Wan et al., 2020 (61)	China	Between January and February 2020	*N* = 137 hospitalized patients; Age [median (IQR)]: 47 (36–55) years; Female sex: 46.70%	–	40^e^/135 (29.6%)	1/40 (2.5%) 1^g^/21 (4.8%)	–	–
Huang et al., 2020 (62)	China	Between December 2019 and January 2020	*N* = 41 hospitalized patients; Age [median (IQR)]: 49 (41–58) years; Female sex: 27%	–	13^c^/41 (31.7%)	5/13^c^ (38%)	–	(12) Median between 8 and 14 days^g^
Wang et al., 2020 (63)	China	Between January and February 2020	*N* = 55 asymptomatic cases; Age [median (lower and upper limits)]: 49 (2–69) years; Female sex: 60%	–	2^f^/55 (3.6%)	–	-	–
Young et al., 2020 (64)	Singapore	Between January and February 2020	*N* = 18 cases in isolation at hospital; Age [median (lower and upper limits)]: 47 (3–73) years; Female sex: 50%	–	2^e^/18 (11%)	–	–	(18) Mean – 8 days^c^
Chen et al., 2020 (65)	China	January 2020	*N* = 99 cases; Age [mean (±*SD*)]: 55 (13.1) years; Female sex: 32%	–	17^g^/99 (17%)	11/17 (64.7%)	–	–
Wu et al., 2020 (66)	China	Between December 2019 and January 2020	*N* = 201 hospitalized patients; Age [median (IQR)]: 51 (43–60) years; Female sex: 36.30%	–	53^c^/201 (26.4%)	44/84 (52.4%)	(201)—median of 13 days (IQR: 10–16 days)	(201)—Median: 2 days (IQR−1–4 days)^g^
He et al., 2020 (67)^*^	China	February 2020	*N* = 54 hospitalized patients (severe and critical); Age (mean): 68 years; Female sex: 37.0%	–	–	26/54 (48.1%)	–	–
Wu et al., 2020 (68)	China	Between January and February 2020	*N* = 80 cases; Age [median (IQR)]: 46.1 (30.7–61.5) years; Female sex: 51.25%	–	–	–	(21)—mean of patients who were discharged after 8 days	-
Sun et al., 2020 (50)	China	Between January and February 2020	*N* = 8 cases (children); Age (lower and upper limits): 2 months to 15 years; Female sex: 25%	–	3^e^/8^c^ (37.5%)	–	(5 severe/critical patients): mean of 18.2 days (*SD*: 4.02 days)	–
Liu et al., 2020 (69)	China	Between January and February 2020	*N* = 15 cases; Age [mean (±*SD*) (lower and upper limits)]: 32 (5; 23–40) years; Female sex: 100% (all pregnant women)	–			(2)—mean: 16 days	–
Cao et al., 2020 (70)	China	Between January and February 2020	*N* = 199 hospitalized patients; Age [median (IQR)]: 58 (49–68) years; Female sex: 39.70%	–	61^b^/199 (30.65%)	–	(199)—Median: 15 days (IQR: 12–17 days)—(with pneumonia) Median: 10 days (IQR: 5–14 days) at ICU	-
Pan et al., 2020 (34)	China	Between December 2019 and March 2020	*N* = 88 cases; Age [median (IQR)]: 56.7 (43.4–66.8) years; Female sex: 51.60%	–	970^e^/32,325 (3%)	–	–	–
Liu et al., 2020 (71)	China	Between January and February 2020	*N* = 73 hospitalized patients; Age [mean (±*SD*)]: 41.6 (14.5) years; Female sex: 43.84%	–	3^e^/73 (4%)	–	–	–
Cao et al., 2020 (72)	China	Between January and February 2020	*N* = 135 cases; Age [mean (±*SD*)]: 48.87 (17.12) years; Female sex: 48.06%	–	2^e^/135 (1.48%)	–	–	–
Simonnet et al., 2020 (73)	France	April 2020	*N* = 124 hospitalized patients at ICU; Age [median (IQR)]: 60 (51–70) years; Female sex: 27%	–	85^h^/ 124^c^ (68.6%)	18/78^c^ (23%)		Time to mechanical ventilation (*n* = 85): 62 cases upon admission; 13 cases at day 1; 4 cases at day 2; and 6 cases within 7 days^c^.
Lian et al., 2020 (46)	China	Between January and February 2020	*N* = 778 cases, divided into elderly group, with 136 cases and mean age of 68.28 years; and non-elderly adult group with 652 cases and mean age of 41.15 years.	–	Group < 60 years: 9^c^/652 (1.38%); 35^g^/652 (5.37%); 1^i^/652 (0.15%); 72^j^/652 (11.04%). Group ≥ 60: 13^c^/136 (9.56%); 23^g^/136 (16.91%);1^j^/136 (0.74%);10^j^/ 136 (7.35%).	–	–	–
Liu et al., 2020 (74)	China	Between January and February 2020	*N* = 56 hospitalized patient, divided into group ≥60 years (*N* = 18), Age [median (IQR)]: 68 (65.25–69.75) years, female sex: 50%; and group < 60 years (*N* = 38), age [median (IQR)]: 47 (35.75–51.25) years; female sex: 33.337% (group < 60 years)	–	4^k^/18 (22.22%) (group ≥ 60) and 2^k^/38 (5.26%) (group < 60 years)	–	–	–
Guan et al., 2020 (75)	China	Between January and February 2020	*N* = 575 hospitalized patients; Age [mean (±*SD*)]: 48.9 (16.3) years; Female sex: 42.70%	–	50^h^/1590 (3.1%); 99^c^/1590 (6.2%)	50/1590 (6.2%)	–	–
Petrie, 2020 (76)	Australia	Between January and February 2020	*N* = 6,606 cases; Age [median (IQR)]: 60.5 (42–72) years; Female sex: 50.00%	–	39^b^/810 (4.8%); 141^c^/810 (17%)	–	–	–
Cai et al., 2020 (77)	China	Between December 2019 and January 31, 2020	*N* = 298 cases; Age [median (IQR)]: 47 (33-61) years; Female sex: 51.34%	–	30^c, h^/298(10.1%)	3/58^d^ (5.2%)	(298)—Median: 20.5 days (IQR: 15–26 days)	–
Bhatraju et al., 2020 (78)	USA	Between February and March 2020	*N* = 24 critical cases; Age [mean (±*SD*)]: 64 (18) years; Female sex: 38%	–	–	12/24^c^ (50%)	(24)—Median: 12 days (IQR: 8–12 days) at hospital; at ICU: 9 days (IQR: 4–14 days) Among survivors: (12) 17 days (IQR: 16–23 days) at hospital; at ICU (survivors): 14 days (IQR: 4–17 days).	–
				–	–		Duration of mechanical ventilation: general 10 days (IQR: 7–12 days); among those who were extubated (*n* = 6/18, 33%) 11 days (IQR: 4–17 days)	–
Jin et al., 2020 (49)	China	Between January and February 2020	*N* = 651, divided into group with gastrointestinal symptoms (GI) (*N* = 21), Age [mean (*SD*)]: 46.14 ± 14.19 years; Female sex: 50%; and asymptomatic group GI (*N* = 577), Age [mean (*SD*)]: 45.09 ± 14.45 years; Female sex: 49.05%	–	With GI symptoms: 5^g^/74^c^ (6.76%); 1^i^/74^c^ (1.35%); 13^j^/74^c^ (17.57%) asymptomatic GI: 12^g^/577^c^ (2.08%); 1^i^/477^c^ (0.17%); 51^j^/577^c^ (8.84%)	1/74^c^ (1.35%) in the group with GI symptoms	–	–
Republic of Korea, 2020 (79)	Korea	January and March 2020	*N* = 94,635; Sex female: 62%	4,212/94,635 (4.45%)	–	–	–	–

a *The proportion of registry of infections at the first moment was estimated at 0.65 (95%CI: 0.60–0.69), that is, 65% of infections were registered/detected, in period 1. This proportion dropped to 14% before travel restrictions and was kept as such throughout the period 2*.

b *Cases who required mechanical ventilation*.

c *Cases who required admission to intensive care unit (ICU)*.

d *Cases reported as critical, with no mentioning of case definition*.

e *Cases who presented shock or who required mechanical ventilation or admission to intensive care unit (ICU)*.

f *Cases of severe respiratory failure, but with no need for monitoring at intensive care unit (ICU)*.

g *Cases who developed acute respiratory distress syndrome*.

h *Critical cases were defined according to WHO guidelines*.

i *Cases who presented shock*.

j *Cases who had liver injury*.

k *Cases with pneumonia severity index (PSI) 4 and 5*.

*ARDS, Acute respiratory distress syndrome; ICU, Intensive care unit; GI, gastrointestinal; 95%CI, 95% confidence interval; IQR, Interquartile range; SD, Standard deviation*.

* *Article in Chinese, only the abstract in English was assessed*.

## Discussion

This study presented a proposal of a rapid literature review method, which identified a set of epidemiological parameters aiming to support construction of predictive models and evidence-based decision-making in view of the COVID-19 pandemic. The syntaxes developed and the rapid review method proposed allowed for identification and synthesizing of all epidemiological parameters of interest in only 40 days. This required the joint effort of researchers and adjustments to the method usually recommended for systematic literature reviews.

Although complex, it is imperative to select good parameters to support mathematical and epidemiological models that predict diseases dynamics in different territories, especially emergent and reemergent epidemics. For COVID-19, the models presented to date are mostly based on local parameters of early stages of the epidemic, as well as the viral behavior of other coronaviruses, such as those causing SARS and MERS outbreaks. Our results show that it is possible to overcome said difficulties by rapidly and systematically gathering evidence produced with different methodologies and in different settings, facilitating identification of parameters that are more suitable to the context and the purpose of the predictive model, improving quality and accuracy of results, and potentially helping territories enhance their COVID-19 preparedness and emergency response.

For all parameters assessed, we found a higher frequency of studies from China. We believe this was due to fact that COVID-19-related cases first emerged in China, which favors a higher number of studies coming from there. Only a few studies were from Europe, the Americas and regions other than Asia, however, with the spread of COVID-19 throughout the world, studies from these regions will be increasingly frequent in the literature, allowing for a more in-depth analysis of other contexts.

One of the parameters most affected by the local context is the reproduction number (R). Cultural habits, control measures in place—such as contact tracing, lockdown or border closures—and the stage of the disease in the territory will directly impact the value and evolution of R (80). Also, limitations concerning data quality and the number of observations have been reported in many studies and may impact estimates. In this sense, we found three outliers in this review. The one with the lowest R (0.48) was developed in South Korea (30) using massive testing, contact tracing and quarantine strategies, in addition to case isolation (81). One of the highest R values (more than 14) was from data on a cruise-ship [i.e., an enclosed population for which, although some restrictive measures were put in place, social distancing was not possible (22, 23)].

Therefore, for construction of predictive models, in order to use the most appropriate R value, it is imperative to understand health systems and their surveillance strategies, as well as consider the social, economic, demographic and cultural contexts of the population for which the estimates are made. It is also worth mentioning that some studies (18, 20, 21, 28, 34) showed a lower R value after restrictive measures were implemented.

The incubation period, infectious period and serial interval are also crucial for understanding the evolution of epidemics. In this regard, there was no wide variation in the incubation period and serial interval among the selected studies, which may contribute to the accuracy of predictive models, however, these results must be consistently confirmed outside of Asia. The scarcity of studies on the transmissible period is another important aspect, and there is a need for new studies estimating this parameter for different populations.

The parameters were mostly extracted for adult, male subjects. Studies suggest that children develop mild symptoms or remain asymptomatic, which hinders case identification, however they play a crucial role in the disease transmission cycle (82). Also, the predominance of males can be explained due to the larger proportion of males in the Chinese population (83). Work conditions of males may also put them at higher risk of exposure to the pathogen, and some health conditions may increase the risk of severe disease (84).

The parameters pertaining to the rate of critical cases among all COVID-19 cases are extremely relevant for managers to anticipate and put in place the logistics and technologies required for critical patient care. Due to the different criteria adopted to define critical cases, it was difficult to establish a homogeneous classification. However, we identified different situations that led to cases being classified as critical, allowing for application of the parameter in predictive models based on the local context or demand. As for the proportion of deaths among critical cases, we also found heterogeneity in the studies. We believe that the criteria used to classify cases as critical may have influenced the way the fatality rate was presented in this clinical classification, leading to inconsistent results.

Variability in case classification is a difficulty in several diseases (85). This heterogeneity is an obstacle in literature reviews and other epidemiological studies, since it precludes head-to-head comparison of research studies. In that sense, we recommend that researchers use a standard classification, based on a protocol such as that of the WHO (86), to standardize case presentation and facilitate data use by other groups. We highlight that in this review, we presented the different classifications of critical cases, allowing modelers and decision-makers to identify parameters according to the context.

The length of hospital stays identified in the studies ranged from one to nearly 3 weeks, and the length of outpatient stay until ARDS onset or ICU admission ranged from immediate up to 2 weeks. This information is relevant so that mathematical models can anticipate the demand for hospital beds, estimated costs and even potential complications arising from long stays, supporting decision-making by managers.

Although the usual method employed in systematic literature reviews is the gold standard (87), particularly due to its minimizing of the risk of bias and ensuring critical and adequate data review, it is time-consuming (88) and usually takes between 6 months and 2 years for completion (89), which limits its use in the current emergency context. By simplifying or omitting components usually included in systematic reviews, rapid literature reviews can be produced faster, although with a higher risk of bias (90).

Thus, this protocol was considered a rapid review because, among the limitations, we highlight the inclusion of only two databases, the language restriction, the non-paired data selection and extraction processes, as well as the absence of a careful evidence quality assessment (90). However, to reduce these limitations, we used sensitive syntaxes in comprehensive databases; all review stages were supervised by experienced researchers with an epidemiology background; meetings were held to standardize concepts and organize the execution of all steps. Also, most parameters were extracted from descriptive observational studies, including cohort studies and case series, using similar methods, leading to relative homogeneity in respect to evidence quality. Furthermore, in terms of limitations, we included studies with different populations—groups restricted to enclosed spaces such as cruise-ships, hospitalized patients and specific professionals, for example—and reviewed data collected using primary and secondary instruments. However, study characteristics are presented in all extraction charts, to make for easier reading.

It should also be noted that some parameters for monitoring the disease progress were not included. These parameters, such as 7-days, or 14-days averages of cases and deaths can be important for health authorities that are using the mathematical models to make decisions regarding the reopening of various societal sectors. However, this rapid review explored the parameters requested by the group of Brazilian mathematical modelers to determine assistance measures, and these parameters, at that time, were not demanded. When replicating this method, the syntax can be easily adapted to obtain these and other parameters, as needed.

Due to the difficulties to define good parameters, we recommend that, when using the data presented in this article, researchers pay attention to disease transmission chains; the contribution of different age ranges to infection strength; the stage of implementation of control measures; and the current and projected health situation in each territory. Modelers must also consider the accuracy of results, assess the number of studies selected, and test uncertainties. We recommend the use of the syntaxes developed and presented in this article when performing new searches to update parameters, contemplating studies conducted in other contexts of time, place, and people, when needed. Also, we believe that these syntaxes can be adapted according to the types of models that are being constructed (e.g., microsimulations, agent-based modeling, systems dynamic modeling, causal inference analysis, economic analysis and other epidemiological and mathematical models) and how impact outcomes are being looked at/predicted.

Knowing the parameters that help understand the dynamics of the SARS-CoV-2 pandemic, such as those presented in this study, allows for modeling of the impact of surveillance and control measures on virus transmission. Mathematical models of transmission estimate the number of infections over time and their consequences, allow for sizing of the resources needed for patient care, and assessment of the impact of non-pharmaceutical interventions (91), supporting decision-making and public policy management.

The rapid literature review methodology used in this study was developed and operationalized in slightly more than 1 month, and showed that it is feasible to rapidly identify and summarize a set of epidemiological parameters in the context of public health emergencies, where an expressive and increasing number of publications can be found. The epidemiological parameters presented here describe information from different scenarios of COVID-19 transmission, disease and deaths and may be used to support predictive models used to estimate the societal impact of the disease, helping decision-makers develop evidence-based preventive measures and ensure preparedness of health systems.

## Data Availability Statement

The original contributions presented in the study are included in the article, further inquiries can be directed to the corresponding author/s.

## Author Contributions

LG, WA, MO, and HP outlined the review. LG, MO, and HP coordinated the review. HP developed the syntaxes. LG conducted literature searches, imported the publications, and removed duplicates. AO, AA, LS, and YM performed study selection and data extraction. MO and HP oversaw data extraction and resolved conflicts. LG, AO, MO, and HP wrote the first version of the manuscript. FS and WA contributed with data analysis and interpretation. MA contributed with data interpretation and manuscript translation. All authors critically reviewed, read, and approved the final version of the manuscript.

## Conflict of Interest

The authors declare that the research was conducted in the absence of any commercial or financial relationships that could be construed as a potential conflict of interest.
